# The associations between current and anticipatory weight-related shame and flourishing in adolescent girls in sport

**DOI:** 10.3389/fpsyg.2025.1535766

**Published:** 2025-06-09

**Authors:** Kristen M. Lucibello, Tara Zeitoun, David M. Brown, Eva Pila, Catherine M. Sabiston

**Affiliations:** ^1^Department of Kinesiology, University of Toronto, Toronto, ON, Canada; ^2^Department of Nutritional Sciences, Temerty Faculty of Medicine, University of Toronto, Toronto, ON, Canada; ^3^School of Kinesiology, Department of Health Science, Western University, London, ON, Canada

**Keywords:** body-related shame, self-conscious emotions, athletes, youth, body image, positive mental health

## Abstract

Flourishing (i.e., positive mental health reflecting positive social relationships and sense of purpose and optimism) is important for experiencing growth, resilience, and functioning – especially in sport. Factors that may limit or potentiate the experience of flourishing in sport need to be understood. For girls involved in sport, weight-related shame may be a critical factor limiting the potential to flourish. The purpose of the present study was to explore current and anticipatory weight-related shame in association with flourishing among adolescent girls. Participants were Canadian girl athletes (*N* = 189) aged 13 to 18 years old (*M* = 15.93, *SD* = 1.22) who had previous or current involvement in organized sport. Girls completed a self-report survey where they reported their current and anticipatory (weight gain or loss) shame and flourishing. A Path model was tested in MPlus. Higher current weight shame [Estimate = −0.41, SE = 0.07, *p* < 0.01] and anticipatory weight loss shame [Estimate = −0.13,. SE = 0.07, *p* = 0.03], were associated with lower flourishing. Anticipatory weight gain shame was not associated with lower flourishing. These results suggest efforts are needed to disconnect the emotion of shame from weight change to foster positive psychological outcomes, such as flourishing, in sport contexts.

## Introduction

Adolescence is a formative developmental period to foster positive mental health and well-being (Vijayakumar et al., 2018). Sport has been identified as an important opportunity to buffer mental illness and promote flourishing during adolescence (Eime et al., 2013). *Flourishing* is defined as high well-being (Keyes, 2002), and reflects an optimal state of functioning, resilience, and growth (Diener et al., 2010; Fredrickson and Losada, 2005). Adolescents who participate in sport report higher flourishing than peers with more sedentary hobbies (Kim et al., 2020). However, girls typically report poorer sport experiences and higher drop-out compared to boys (Canadian Women and Sport, 2020; Slater and Tiggemann, 2011), and the gendered influences that contribute to these patterns may impact not only sport-related experiences, but also more general well-being indicators such as flourishing.

Accumulating evidence suggests that the girls sport context can be appearance-focused, comparative, and evaluative of the body (Willson and Kerr, 2022; Vani et al., 2020), with a particularly strong focus on body weight. Girls have reported pressure from their coaches to lose or maintain their weight (Coppola et al., 2014; Voelker and Reel, 2015), and commentary about their weight from family, teammates, spectators, or opponents in sport (Lucibello et al., 2021a; Murray et al., 2022; Porter et al., 2013). Girls also perceive the importance of meeting a sport-related weight and body ideal, and that athletes can be favored based on weight as opposed to performance (de Oliveira et al., 2021; Lunde and Gattario, 2017). Critically, the perpetuation of body weight importance, stringent weight ideals, and weight management in girls sport may diminish flourishing in athletes by fostering body-related shame.

Body-related self-conscious emotions, an affective domain of body image, represent positive and negative emotions contextualized to the body’s appearance (Castonguay et al., 2014; Sabiston et al., 2020). Body-related shame is an intense negative self-conscious emotion elicited when one attributes perceived deviance from societal body standards to a flawed global self (Sabiston et al., 2010; Tracy and Robins, 2006). Girl athletes report body-related shame contextualized to their sport context (Coppola et al., 2014; Huellemann et al., 2023) and increases in body-related shame have been observed over time in girl athletes (Sabiston et al., 2020). Critically, higher current body-related shame has been associated with lower flourishing in girl athletes (Gilchrist et al., 2021), suggesting the body-related shame fostered in response to the weight-focused and evaluative sport context may impact flourishing.

However, two important limitations to the above evidence warrant consideration. First, examination of the association between body-related shame and flourishing has been limited by a focus on global body appearance as opposed to weight-specific shame that may be uniquely impacted by the weight-centric girls sport environment. Second, little is understood about *anticipatory* shame specific to weight changes in girl athletes. Anticipatory shame describes how ashamed an individual perceives they *would* feel in response to a situation that could occur (e.g., shame in response to weight gain). Anticipatory shame may be particularly relevant to girl athletes, as Objectification Theory highlights that girls are generally socialized to anticipate how others will view and evaluate their bodies (Fredrickson and Roberts, 1997). These tendencies for objectification and subsequent shame may be exacerbated within the highly evaluative girls sport context which contains explicit weight and body commentary and ideals (Lucibello et al., 2021a; Murray et al., 2022). Being in a normalized weight-focused environment while witnessing favoritism and bias toward athletes of a certain weight may make girl athletes vulnerable to high anticipatory shame, due to regular messaging about how weight changes could negatively impact their sport experiences and performance.

Importantly, anticipatory emotions have unique influences on behavioral and psychological outcomes above and beyond current emotions (Troop et al., 2006). Anticipatory body-related shame in response to weight gain has been associated with numerous maladaptive outcomes, including depressive symptoms, excessive exercise, fasting, dieting, body checking, binge eating, and fear of weight gain (Troop, 2016; Troop and Redshaw, 2012). While anticipatory emotions are often investigated as motivators of goal-directed behaviors (Pila et al., 2021) they may also be related to aspects of well-being and flourishing. Given that sport is considered a channel to promote flourishing in adolescence (Eime et al., 2013), understanding whether anticipatory weight-related shame may diminish flourishing is an important consideration for girl athletes.

The present study examined (i) mean differences in current and anticipatory weight-related shame, and (ii) the associations among current weight-related shame, anticipatory weight-related shame, and flourishing in adolescent girl athletes. Based on theoretical tenets and empirical evidence (Fredrickson and Roberts, 1997; Gilchrist et al., 2021; Troop, 2016), it was hypothesized that higher (i) current weight shame and (ii) anticipated weight gain/loss shame, would be associated with lower flourishing.

## Methods

### Participants and procedures

Data were drawn from a prospective longitudinal cohort study of adolescent girl athletes enrolled in organized team-based sport, recruited at an average age of 14 from the Greater Toronto Area and followed for three additional years (Sabiston et al., 2020). Study advertisements were relayed to coaches of girls sport teams and administrators at local sport organizations on “the effect of playing organized sport on the mental, physical, and social well-being of adolescent girls.” Any adolescent enrolled on a girls team that coaches or administrators contacted was eligible to participate. Following participant assent and parental consent, participants completed a 20–30-min survey in a group setting supervised by trained research assistants. Participants were compensated with a gift card ($10 CAD) and given the option to be contacted for yearly follow-up online surveys. Data for the current analysis (*n* = 189) is from the third time point, approximately 2 years following baseline when the girls were an average of 16 years old, as this wave is when measures of anticipatory weight-related emotions were included. This sample size is consistent with sample size estimates for path modeling which suggest at least 10 participants per parameter (Bentler and Chou, 1987). All study procedures were approved by the University of Toronto research ethics board.

### Measures

#### Demographic characteristics

Participants reported their age, ethnicity, sport status, height (ins) and weight (lbs), to calculate body mass index (BMI)-for-age growth percentiles (De Onis and Onyango, 2008).

#### Weight-related shame

The 15-shame items from the 30-item Bodily Pride Shame Scale (BPSS) measured behavioral, affective and attitudinal aspects of current and anticipated weight shame (Troop, 2016). The BPSS has three subscales: current weight, anticipated weight gain (~ 15 lbs), and anticipated weight loss (~ 15 lbs). Each subscale contains five items (e.g., “I am/would feel ashamed of my body”) with responses ranging from 1 (*not at all true of me*) to 10 (*completely true of me*). The items in each subscale are identical except for the time frame (current, anticipated) and weight change direction (loss, gain). Higher mean subscale scores indicate greater weight shame. Adequate internal consistency was noted for current weight shame (*α* = 0.82), anticipatory weight gain shame (*α* = 0.90), and anticipatory weight loss shame (*α* = 0.83).

#### Flourishing

The 8-item Flourishing Scale (Diener et al., 2010) was used to measure participants’ self-perceived positive functioning and success in areas ranging from relationships, self-esteem, purpose, and optimism (e.g., “my social relationships are supportive and rewarding”). Items are answered on a 7-item Likert scale ranging from 1 (*strongly disagree*) to 7 (*strongly agree*), with higher mean scores reflecting higher levels of flourishing (*α* = 0.90).

### Data analysis

Normality was assessed for continuous variables, and descriptives, Cronbach’s alpha, and bivariate correlations were computed. Paired sample t-tests compared the mean differences in shame (current, anticipatory gain, anticipatory loss), and a path model with maximum likelihood robust estimation was tested in *MPlus* Version 8.5 (Muthén and Muthén, 2019). The hypothesized positive correlation between current shame, weight gain shame, and weight loss shame, was specified. The empirically supported variables of body mass index (continuous, age- and sex-based percentile; De Onis and Onyango, 2008) and current sport participation (yes/no) were selected a-priori and included as covariates (Goldstein et al., 2021; Kim and Jang, 2018). Model fit was assessed using comparative fit index (CFI; values ≥ 0.95), standardized root-mean-square residual (SRMR; ≤ 0.08), and root mean square error of approximation (RMSEA; < 0.10) (Browne and Cudeck, 1993; Hu and Bentler, 1999).

## Results

Demographic characteristics, means, standard deviations, and bivariate correlations are presented in Table 1. Girls were an average of 15.93 years (*SD* = 1.22 years), while the mean BMI-for-age percentile was 44.9th (*SD* = 29.3rd percentile). Participants predominantly identified as White (*n* = 150, 74.6%) followed by another ethnicity (*n* = 11, 6%), multiple ethnicities (*n* = 9, 4.5%), Black (*n* = 7, 3.5%), Chinese (*n* = 7, 3.5%), and South Asian (*n* = 6, 3.0%). Most participants (*n* = 127, 67%) were participating in sports at the third time point; girls most commonly reported soccer as their primary sport (*n* = 113, 56.2%), followed by hockey (*n* = 24, 11.9%), swimming (*n* = 21, 10.4%), gymnastics (*n* = 8, 4.0%), and skiing (*n* = 6, 3.0%). All other primary sports (*n* = 12, e.g., dance, volleyball) were endorsed by under 3% of the sample.

**Table 1:** Score ranges, means, standard deviations, and bivariate correlation coefficients of main study variables (*n* = 189).

Variable	M (SD)	Range	1	2	3	4	5	6	7	8
1. Age	15.93 (1.22)	13–18	–							
2. BMI-z	44.92 (29.25)	3–99	0.13	–						
3. Ethnicity	N/A		0.08	−0.02	–					
4. Sport status	N/A		−0.16*	−0.17*	0.07	–				
5. Current shame	4.13 (2.06)	1–10	0.16*	0.36**	−0.14	−0.33**	–			
6. Gain shame	6.51 (2.67)	1–10	0.23**	0.33**	0.02	−0.18*	0.60**	–		
7. Loss shame	2.08 (0.90)	1–10	0.08	−0.19**	0.01	0.00	0.15*	−0.13	–	
8. Flourishing	5.75 (0.88)	1–8	0.08	−0.04	0.19**	0.24**	−0.43**	−0.23**	−0.19**	–

SD = standard deviation; BMI-z = body mass index percentile; ethnicity [0 = Black, Indigenous, and People of Color, 1 = white], sport status [0 = sport drop out, 1 = continued sport participation]; *p < 0.05, **p < 0.01.

Participants reported significantly higher anticipatory weight gain shame (*M* = 6.51, *SD* = 2.67) than current shame (*M* = 4.00, *SD* = 2.06) [*t*(376) *=* −9.6*, p* < 0.01], and lower anticipatory weight loss shame (*M* = 2.08, *SD* = 0.89) than current shame [*t*(376) *=* 12.50*, p* < 0.01].

**Figure 1:**
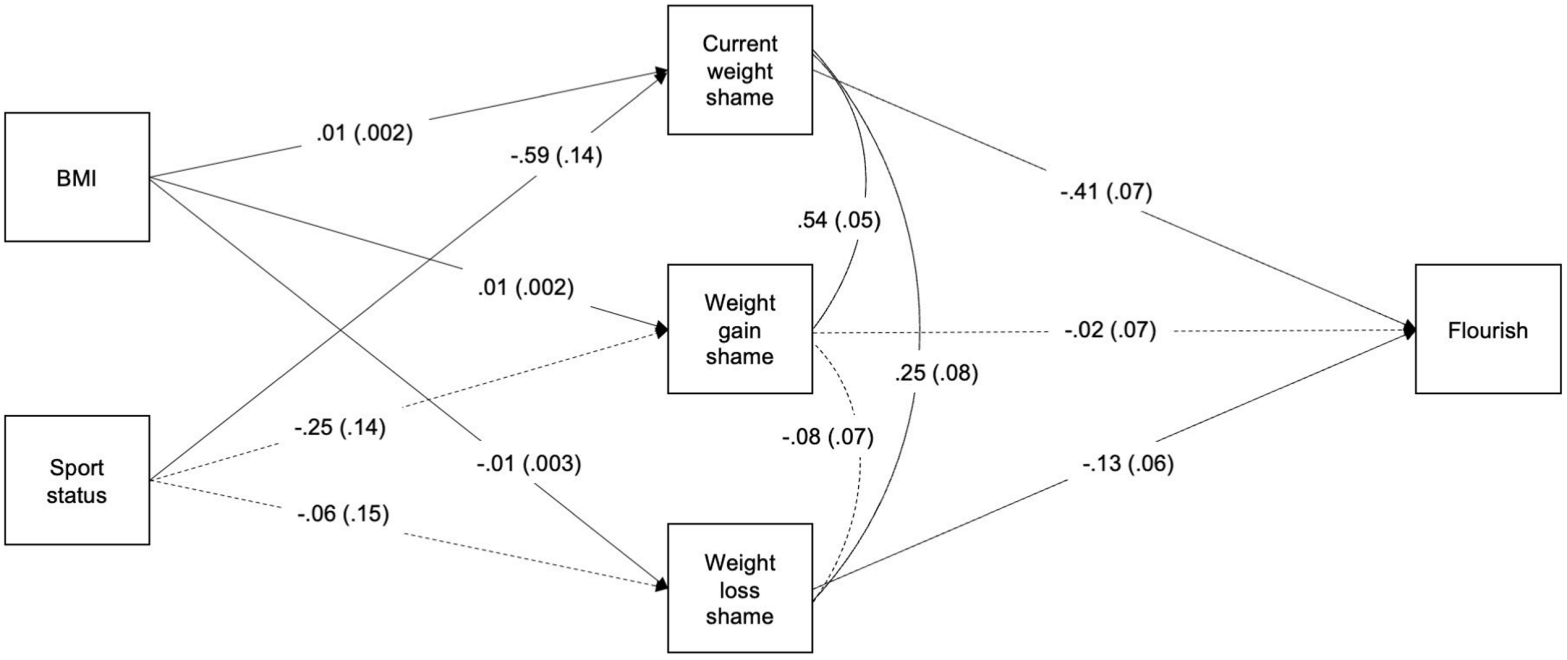
Path model representing the associations among current weight-related shame, anticipatory weight-related shame, and flourishing, after controlling for sport status and BMI. BMI = body mass index (age and sex-percentile), sport status = 1 (yes currently participating in sport), 2 (no longer participating in sport), flourish = flourishing. Significant paths indicated by solid arrows (*p* < 0.05), nonsignificant paths indicated by dashed arrows.

The final path model demonstrated adequate fit [*χ*^2^(2) = 5.28, *p* = 0.07; CFI = 0.98; SRMR = 0.03; RMSEA = 0.09 (90% CI = 0.00, 0.19)], with full path models results presented in Figure 1. Higher current weight-related shame [Estimate = −0.41, SE = 0.07, *p* < 0.01] and anticipatory weight loss shame [Estimate = −0.13,. SE = 0.07, *p* = 0.03], but not anticipatory weight gain shame, were associated with lower flourishing.

## Discussion

The current study examined the associations between current and anticipatory shame and flourishing. Consistent with previous evidence (Troop, 2016), girls reported significantly higher levels of anticipatory weight gain shame than current weight shame or weight loss shame. However, only higher current shame and anticipated weight loss shame were associated with lower flourishing. This study extends previous research by utilizing weight-contextualized measures of shame to capture the potential body image ramifications of the weight-focused nature of girls’ sports. These findings also extend understanding of the impact of anticipatory emotions on flourishing and offer further support for larger-scale changes to girls’ sport (i.e., mandating weight neutral language, sport leaders emphasizing body functionality as opposed to appearance, body diverse imagery; Koulanova et al., 2021) that may mitigate current and anticipatory weight-related shame.

Although low average levels were reported relative to current and anticipatory weight gain shame, higher anticipated shame in response to weight loss was associated with lower flourishing. Given the focus on current emotional experiences in research involving girl athletes, these findings suggest that broadening assessments of body image to include anticipatory emotions may provide a more nuanced understanding of how the girls sport context contributes to negative body image, as well as how body image relates to psychological outcomes in girl athletes. Anticipatory weight loss shame relating to flourishing may indicate adolescent girl athletes’ investment in maintaining a particular weight within sport, consistent with messaging of weight maintenance from some coaches (Coppola et al., 2014; Lucibello et al., 2021a). Identifying girls who may be more vulnerable to anticipatory weight loss shame may be a useful approach to mitigating feelings of anticipatory shame. For example, self-objectification was associated with anticipatory shame in a previous reporting of this sample (Pila et al., 2021). Similarly, internalized weight stigma has been associated with higher current body-related shame in young women (Lucibello et al., 2021b). Anticipating shame in response to weight loss may reflect greater investment in body weight and appearance (Cash et al., 2004), or internalization of weight norms and societal standards (Tiggemann, 2011), which may also relate to lower flourishing. Therefore, more complex pathways that may explain the association between anticipatory shame and flourishing should also be investigated. For example, anticipatory shame promotes engagement in negative behaviors such as fasting and compulsive exercise (Troop and Redshaw, 2012), which may be an indirect pathway that reduces flourishing over time. Longitudinal research is imperative for understanding the interpersonal factors that predict anticipatory shame and the mechanisms through which anticipatory weight loss shame may reduce flourishing in girl athletes.

Although the high levels of anticipatory weight gain shame are consistent with the pervasive fat bias and weight stigma that exist within girls sport (Coppola et al., 2014; Huellemann et al., 2023; Sabiston et al., 2020), anticipatory weight gain shame was not significantly associated with flourishing among girl athletes, while controlling for covariates and accounting for current and anticipated weight loss shame. However, it is important to acknowledge the limitations of the measure in understanding anticipatory shame for weight gain among adolescent girls. Anticipatory weight gain shame was the highest type of shame reported; a high and relatively ubiquitous emotional response may suggest the 15-pound weight gain scale is too drastic to capture nuance or variability in this age group relative to young adult women (Troop, 2016). The bivariate correlation was also significant between anticipatory weight gain shame and flourishing in the expected direction. Therefore, future research that investigates anticipated shame in response to more subtle or variable changes in weight gain is integral for understanding adolescent girls’ experiences of weight gain shame within the weight-centric and fat phobic sport context.

Consistent with previous literature examining global appearance emotions as well as general negative emotions as contributors to reduced flourishing (Fredrickson and Losada, 2005; Gilchrist et al., 2021), higher current weight-related shame was related to lower flourishing. While body-related shame has been identified as an important target for improving sport experiences for girl athletes (Pila et al., 2020) these findings also support the importance of considering their influence on psychological factors outside of the sport context.

While causation cannot be inferred due to the cross-sectional design, these findings suggest that reducing current and anticipatory weight-related shame in girl athletes may foster and optimize their potential for flourishing. This may be done by reducing weight- and body-related comments and pressures within the sport environment (Koulanova et al., 2021). The pervasiveness of weight ideals, commentary, and pressures in girls sport (Coppola et al., 2014; Lucibello et al., 2021a) has been noted as a significant distraction for girl athletes (Lauer et al., 2018) and can contribute to poorer sport experiences and drop out (Pila et al., 2020). Negative sport experiences may in turn foster shame, potentially reducing flourishing; notably, a reduction in flourishing may lead to lasting negative health behaviors (e.g., disordered eating; Zemestani et al., 2021) and sport drop out (Sabiston et al., 2020).

There are some study limitations to address. First, there are additional negative body-related self-conscious emotions (e.g., guilt, embarrassment) that could not be investigated due to a lack of validated anticipatory measures. Additionally, the girl athletes were predominantly White, limiting statistical power for examining associations across race or ethnicity. Due to limited sample size investigation into differences based on sport (i.e., hockey vs. swimming) and sport features (i.e., aesthetic vs. non-aesthetic, team vs. individual sport; Abbott and Barber, 2011; De Bruin et al., 2007) could also not be explored. Similarly, by only examining girls in sport as opposed to comparing mean levels and associations with girls not in sport, the impact of the sport environment in fostering shame cannot be determined. Lastly, the cross-sectional data warrants further investigation into the associations among anticipatory emotions and flourishing over time.

In conclusion, this study provides preliminary support for the associations among current and anticipatory weight loss shame and flourishing in girl athletes. Future longitudinal and experience sampling studies will provide important insight into the mechanisms, moderators, and outcomes of current and anticipatory shame, contributing to the enhancement of girls’ sport experiences and well-being.

## Data Availability

Data is only available upon reasonable request to the corresponding author.
